# Activity of Haliscosamine against *Fusarium oxysporum* f.sp. *melonis*: *in vitro* and *in vivo* analysis

**DOI:** 10.1186/s40064-015-0797-x

**Published:** 2015-01-13

**Authors:** Belakssem El Amraoui, Jean François Biard, Fatima Ez-Zohra Ikbal, Majida El Wahidi, Mostafa Kandil, Mohammed El Amraoui, Aziz Fassouane

**Affiliations:** Faculté Polydisciplinaire de Taroudant, Université Ibn Zohr, Taroudant, Maroc; Laboratoire Contrôle Qualité en Bio-Industrie et Molécules Bio-Actives, Faculté des Sciences, Université Chouaib Doukkali, BP 20, El Jadida, 24000 Maroc; Laboratoire MMS, Faculté de Pharmacie Université de Nantes, Nantes, France; Laboratoire de Physiologie végétale et phytopathologie, Faculté des Sciences, Université Chouaib Doukkali, BP 20, El Jadida, 24000 Maroc; Laboratoire d’Anthropogénétiques et Biostatistiques, Faculté des Sciences, Université Chouaib Doukkali, BP 20, El Jadida, 24000 Maroc; Ecole Nationale du Commerce et de Gestion ENCG de Settat, Settat, Maroc

**Keywords:** *Fusarium oxysporum*, Porifera, Haliscosamine, Marine sponges, *Haliclona*

## Abstract

Marine sponges are a potential source of new molecules with diverse biological activities. We have previously isolated a sphingosine derivative, (9Z)-2-amino-docos-9-ene-1,3,13,14-tetraol (Haliscosamine) from the Moroccan sea sponge *Haliclona viscosa*. The aim of this study was to test Haliscosamine *in vitro* and *in vivo* for its antifungal activity against *Fusarium oxysporum* f.sp. *melonis* causing fusarium wilt of melon.

Overall, *in vitro* test showed that haliscosamine has a similar effect as DESOGERME SP VEGETAUX®. In addition, *in vivo* showed a significant effect against *Fusarium oxysporum* f.sp. *melonis*. Taking to gather, our results suggest that haliscosamine constitutes a potential candidate against *Fusarium oxysporum* f.sp. *melonis* and the possibility to use in phytopathology.

## Introduction

Agriculture is an important economic sector in Morocco ; itemploys about 40% of the nation's workforce. The harvest of melon is popular in Morocco; it is found throughout the country. Moreover, Morocco is the 12^th^ largest exporter to export 55,000 tons of melon in 2009 (El Ouafi 2009). However, diseases that still cause problems in melon, are especially Fusarium followed by powdery mildew and bacterial blight (Messiaen et al. 1991). Fusarium wilt caused by *Fusarium oxysporum* f.sp. *melonis* (FOM), is a major disease affecting melon production in the province of El Jadida (Morocco) and causes important economic losses in this area. Thus, the suppression of this pathogen is considered urgent and a big challenge for this type of agriculture. Indeed, preventive treatment using chemical pesticides is the only way to fight these fungi. However, chemical pesticides sprayed into the air or discharged into the soil can be harmful to the environment and to humans. Biological antifungal may be an alternative. Furthermore, Marine sponges are a potential source of new biological compounds with diverse biological activities (Acosta and Rodriguez 1992; Baslow and Turlapaty 1969; Akiyama et al. 2009; Bao et al. 2007a; Bao et al. 2005; Bao et al. 2007b). In Morocco, few studies are carried out about Moroccan sponges with an important biological material for the isolation of new molecule (El Amraoui et al. 2014b; EL Amraoui et al. 2014a; El Amraoui et al. 2013; El Amraoui et al. 2010; El-Wahidi et al. 2011; El-Wahidi et al. 2013). Haliscosamine isolated from the Moroccan marine sponge *Haliclona viscosa* is a new derivative of sphingosine with an original molecular structure ((Z)-2-amino-docos-9- ene-1,3,13,14-tetraol) (El Amraoui et al. 2013). This compound is active against human pathogenic yeasts, *Candida albicans*, *Candida tropicalis* and *Cryptococcus neoformans* (El Amraoui et al. 2013).

To put it briefly, the aim of this study was to test the antifungal activity *in vitro* and *in vivo* of haliscosamine against *Fusarium oxysporum* f.sp. *melonis*.

## Results and discussion

**Figure 1:**
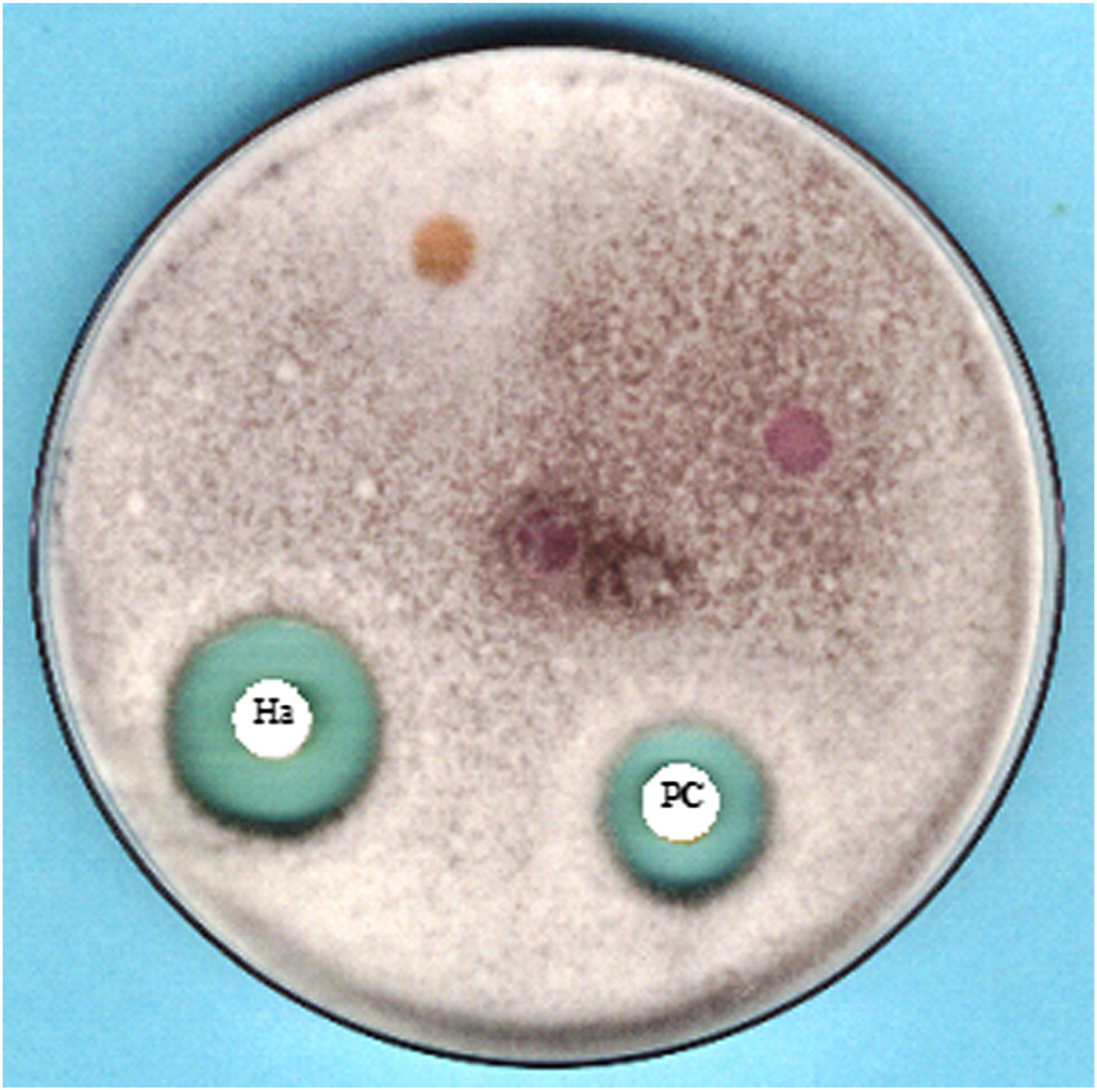
**Example of** ***in vitro*** **antifungal activity of Haliscosamine (Ha) and** ***DESOGERME SP*** **(PC) against** ***Fusarium oxysporum*** **f.sp.** ***melonis*** **.**

Antifungal *in vitro* test *has* shown that Haliscosamine is more active than DESOGERME SP against FOM with inhibition diameters of 21 mm and 19 mm respectively as illustrated in Figure 1. Haliscosamine showed fungicidal activity against *FOM*.

**Figure 2:**
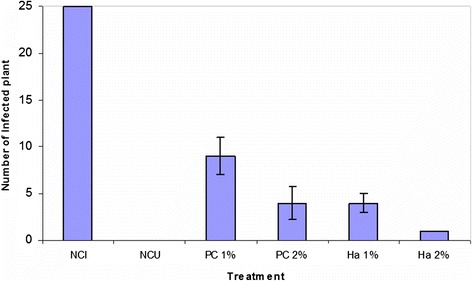
**Average infected seedlings according to the** ***in vivo*** **treatment (NCI: Negative control infested, NCU: Negative control Uninfested, PC: positive control, Ha: Haliscosamine).**

Interestingly, *in vivo* result showed that the average number of infected seedlings is significantly lower than the average number of infected seedlings in the positive control (Figure 2). *Fusarium oxysporum* f.sp. *melonis* had been suppressed by Haliscosamine treatment in infected plant with different concentrations (1% and 2%). This result indicates that the inhibition of *Fusarium* was a concentration-dependent manner of Haliscosamine (Figure 2).

**Figure 3:**
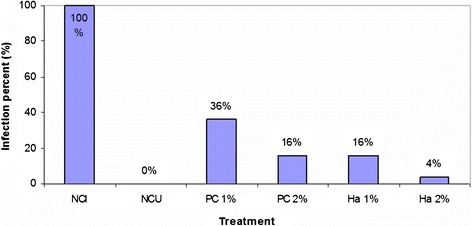
**Percentages of seedlings infected with the pathogen in each treatment and in the controls (NCI: Negative control infested, NCU: Negative control Uninfested, PC: positive control, Ha: Haliscosamine).**

Figure 3 shows the percentages of seedlings infected with the pathogen in each treatment and in the controls. No seedling (0%) of the negative control uninfected (NCU) has presented infection while all seedlings (100%) of the negative control infected (NCI) were infected.

The analysis of variance (ANOVA1) show highly significant (P < 1‰) factor treatment (intergroup variation). Comparison of means by Duncan's test helped highlight homogeneous groups at the 5%. Haliscosamine 1% has an effect similar to DESOGERME SP 2% on the inhibition of *FOM*. In comparison with the positive control, Haliscosamine has a significant inhibitory effect on the disease development.

The genus of *Haliclona* sponges are known for their high chemical various secondary metabolites with interesting biological activities (Faulkner 2002) including the antifungal (Barrett et al. 1996; Clark et al. 2001; Wattanadilok et al. 2007), antileishmanial (Dube et al. 2007), antioxidant (Regoli et al. 2004), cytotoxic (Erickson et al. 1997; Fusetani et al. 1989) and other activities (Hattori et al. 1998; Randazzo et al. 2001; Lakshmi et al. 2009; Roper et al. 2009).

Until now, the research that has been conducted on *H. viscosa,* led to the isolation of a number of alkaloids (Timm et al. 2010). Fuestani et al. (1989) have isolated two cytotoxic compounds, Haliclamine A and B from *H. viscosa*. Volk and Kock 2003 isolated viscosamine, two forms of viscosaline have recently been isolated (Schmidt et al. 2012). Two other alkaloids, haliclamine C and D, were isolated from *H. viscosa* (Volk et al. 2004). In a recently published work, *Haliclona viscosa* has shown significant antifungal activity against plant pathogenic fungi from *Fusarium*, *Botrytis* and *Penicillium* genus (El Amraoui et al. 2014b).

Haliscosamine isolated from *Haliclona viscosa* sponge, has a strong antifungal activity with a wide spectrum. It is active against human pathogenic yeasts (*Candida albicans* ATCC 10231, *Candida tropicalis* R2 CIP 1276.81 and *Cryptococcus neoformans* ATCC 11576) (El Amraoui et al. 2013) and against a very resistant phytopathogenic fungus (*Penicilium digitatum*) (EL Amraoui et al. 2014a).

Sponges, since a long time have been a major source of new biomolecules and they are still the inexhaustible source of new products with different biological activities; they can be used in various areas. Moroccan sponges are little studied, and yet they constitute a new biological material for researchers who are limited to medicinal plants and beach’s invertebrates and algae.

## Conclusion

Haliscosamine isolated from the Moroccan sponge, *Haliclona viscosa* showed *in vitro* fungicidal activity against redoubtable-phytopathogenic fungi. The *in vivo* studies of this product against Fusarium wilt showed promising results. Haliscosamine can be studied more effectively (open-field activity and toxicity) to see the possibility of its use as a biopesticide.

## Materials and methods

### Phytopathogen strains

The phytopathogen strain of the fungus *Fusarium oxysporum* f.sp. *melonis*, Fom 20474 CECT (Coleccion Espanola de Cultivos Tipo) was used in this study (Suárez-Estrella et al. 2007; Suárez-Estrella et al. 2004).

### Haliscosamine

Haliscosamine is an antifungal isolated from the Moroccan marine sponge *Haliclona viscosa.* It is a new derivative of sphingosine with an original molecular structure ((Z)-2-amino-docos-9- ene-1,3,13,14-tetraol) and it is active against human pathogenic yeasts, *Candida albicans*, *Candida tropicalis* and *Cryptococcus neoformans*. Haliscosamine used in this study, was isolated as described previously (El Amraoui et al. 2013).

### DESOGERME SP VEGETAUX®

DESOGERME SP VEGETAUX® (LAKORALE, Morocco), used in this study as a positive control, is an algaecide, fungicide and bactericide product used in Morocco both to remove algae, fungi and bacteria in irrigation systems and also to disinfect soil. It consists of 20 g/L of polyhexamethyle bioguanidine hydrochlorique and 50 g/L of N-alkyl dimethyl benzyl ammonium chloride (EL Amraoui et al. 2014a).

### *In vitro* antifungal activity

This test uses Potato Dextrose Agar (PDA) as medium [Difco]. Conidial suspension was prepared from a 5-dold fungal culture (FOM culture was covered with 10 ml of distilled water and then scraped with a sterile glass rod; spores were recovered after filtration on sterile wool cotton) and adjusted with Malassez’s cellule in sterile water in order to obtain a final concentration of 10^5^ conidia/mL. Each disk 6 mm in diameter received 20 μg of haliscosamine (20 μL of pur haliscosamine at 1 mg/mL in CH_2_Cl_2_ [Difco] were added to each cellulose disc) and then dried and placed on previously inoculated PDA medium. Plates were first kept at 4°C for at least two hours to allow the diffusion of chemicals, and then incubated at 28°C. Inhibition was scored by the absence of any contact between the discs and fungi after 48 h of incubation then inhibition zones were measured. Standard disks of the DESOGERME SP VEGETAUX® (20 μl/disc), served as the positive antifungal controls. All the assays were carried out in triplicate.

To determine whether the haliscosamine has fungistatic (temporary inhibition) or fungicide (permanent inhibition) effect on FOM, agar cylinder was cut out from inhibition zone and placed on the PDA medium and revival of their growth was observed. The fungicidal effect was where there was no growth after additional nine days of incubation at 25°C; whereas, a fungistatic effect was where temporary inhibition of mycelial growth occurred (Askarne et al. 2012).

### *In vivo* antifungal activities of Haliscosamine and DESOGERME SP VEGETAUX® against Fusarium wilts of melon

Haliscosamine was assayed in a greenhouse to determine if it possessed the ability to suppress Fusarium wilt of melon plants. In these tests, two DESOGERME SP VEGETAUX® solutions, 1% and 2% were used as positive control.

Initially, seedlings of charentais melon (No resistance to Fusarium wilt) were planted in 20-cm-diameter pots containing 2.5 L of sterile substrate [Plantaflor PROFI TYP3]. The haliscosamine was dissolved in DMSO and solutions of 1% and 2% were prepared in the irrigation water. Then, the pots were irrigated for two days by each of these solutions. On the third day, a 7-d-old FOM culture grown in potato dextrose broth (PDB) was added to the pots containing plants. Pathogen inoculum which consisted of a mixture of conidia and chlamydospores, was added to the potting mix at a rate of 1000 propagules/g of substrate (Suárez-Estrella et al. 2007). Each treatment consisted of five replicate pots of five plants per pot. Disease was monitored for 6 weeks. Stem sections of all seedlings were destructively harvested and surface disinfected in 0.5% household bleach (0.0026% sodium hypochlorite) and placed on PDA to confirm the presence of the pathogen. Results were shown as the total percentage of seedlings infected with the pathogen.

Two negative controls (without any prior treatment) were used:

- Negative control infested (NCI): All plants of NCI were infested with *FOM* without any prior treatment.
- Negative control Uninfested (NCU): No plants of CNU has infected or treated.

### Statistical analysis

One-way analysis of variance (ANOVA) was used to highlight the effect of treatment on the development of the plant pathogen. Averages of infected plants of different treatments were compared by Duncan test. P-value <0.05 was considered as a significant difference. Statistical analysis of data was performed using the SPSS software package 10.0 (SPSS Inc. USA).
